# Pneumorrhachis Resulting in Transient Paresis after PICC Line Insertion into the Ascending Lumbar Vein

**DOI:** 10.7759/cureus.833

**Published:** 2016-10-17

**Authors:** Russell Payne, Emily P Sieg, Arabinda Choudhary, Mark Iantosca

**Affiliations:** 1 Department of Neurosurgery, Penn State Hershey Medical Center; 2 Pediatric Radiology, Nemours Alfred I. duPont Hospital for Children

**Keywords:** pneumorrhachis, ascending lumbar vein, intraspinal air, picc

## Abstract

Obtaining intravascular access in the neonatal intensive care unit (NICU) is not only critical but also technically challenging. Malposition of the catheter tip is a known and well-documented complication. Specifically, peripherally inserted central venous catheter (PICC) line insertion into the ascending lumbar vein can lead to neurological dysfunction and, in some cases, even death.

We present the first reported case of pneumorrhachis (PR) following PICC line insertion into the ascending lumbar vein. Our patient presented with lower extremity weakness and imaging confirmed the presence of air within the spinal canal. After conservative treatment, the strength deficit resolved and subsequent imaging revealed resolution of the air within the spinal canal.

Insertion of central venous catheters into the ascending lumbar vein is a well-documented complication that can lead to neurologic injury and even death. This should be considered in the evaluation of any neonate presenting with an abnormal neurological examination or unexplained change in exam after line insertion.

## Introduction

Obtaining reliable and sustainable venous access is critical for patients in the NICU. These patients often require administration of medications that can damage small vessels due to their non-physiologic pH or high osmolality as well as early and aggressive total parenteral nutrition (TPN). PICC lines provide long-term venous access with increased flow rates and caloric concentration compared to peripheral intravenous (IV) therapies. These lines are placed by using ultrasound to access a peripheral vein, placing an introducer sheath and manipulating the catheter to a central position. PICC lines have become prevalent due to their rapid and simple insertion and need for only mild sedation but are not without risk. In fact, a large prospective cohort study by Costa, et al. found that 37.2% of PICC lines were removed due to complications [1]. Reports of serious complications related to catheter tip position are common. They include accounts of misplacement or migration into cardiac chambers, subclavian veins, internal jugular veins, renal veins, superficial abdominal veins, and ascending lumbar veins. Additionally, multiple cases of neurological complications related to placement of the catheter into the ascending lumbar vein have been reported.

The ascending lumbar vein originates from the common iliac vein and drains the epidural venous plexus. This anatomic configuration allows for the potential placement of central venous catheters within or immediately proximal to the epidural venous plexus when the ascending lumbar vein is inadvertently cannulated. Malposition of the PICC line tip into the ascending lumbar vein leading to serious neurological compromise in neonates has been well described in the literature [2-3]. Some authors have reported perforation of the venous system leading to extravasation of intravenous fluids into the spinal canal. In some reported cases, this was confirmed via lumbar puncture and removal of fluid consistent with TPN [4]. In those experiencing neurological complications due to aberrant PICC line insertion, the documented appearance of symptoms occurred between one and 11 days after their placement. The presentation was varied with some infants initially exhibiting lethargy and others experiencing seizures or lower extremity weakness. Some infants suffered permanent neurological damage in the form of neurogenic bladder or flaccid paraplegia [5]. A case series of six patients with inadvertent insertion of a central venous line into the epidural venous plexus via the ascending lumbar vein reported by Lavandosky et al. [6] showed that there are several warning signs to be cognizant of when inserting a central line that may indicate inadvertent insertion into the ascending lumbar vein: 1) no aspiration of blood after catheter placement, 2) lateral “hump” deviation of catheter at L4-L5 on abdominal flat plate radiograph, and 3) superimposed appearance of catheter tip on the spinal canal on abdominal cross-table lateral radiograph [6]. We report here a complication of inadvertent placement of a PICC line into the ascending lumbar vein that has not been previously documented in the literature. In the following paragraph we discuss a case of pneumorrhachis after PICC line insertion in a neonate. Informed consent was obtained from the patient's parent/guardian for the treatment.

## Case presentation

**Figure 1:**
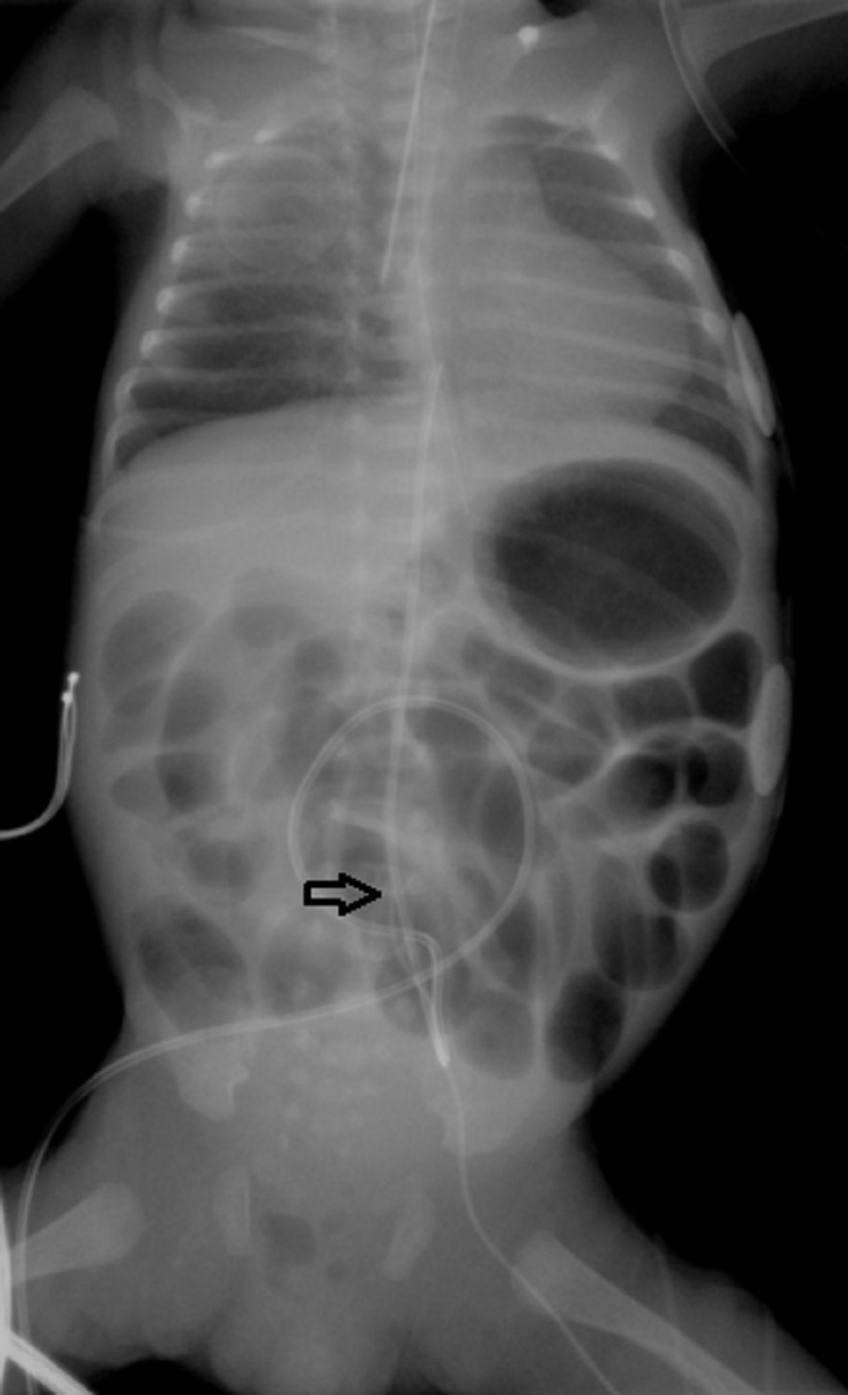
Supine Abdominal Radiograph Demonstrating the left femoral PICC line terminating possibly in a left lumbar vein (Arrow)

A three-day-old premature female infant born by emergent caesarean section at an outside institution was transferred to our NICU for evaluation of decreased lower extremity movement after insertion of a PICC line. The infant was born at 28 weeks due to maternal preeclampsia and decreased fetal movement. Her Apgar scores were six and nine. After birth, an umbilical artery catheter was placed. A PICC line was then placed in the left leg on day of life (DOL) one (Figure 1). After line placement, the infant exhibited minimal movement bilaterally in the lower extremities. Multiple imaging studies including an echocardiogram, a cranial ultrasound and plain film X-rays to evaluate line placement were performed on DOL two in an attempt to elucidate the etiology of this new onset of weakness. The PICC line was found to be malpositioned likely tracking into one of the lumbar veins. It was, therefore, removed on DOL three. Subsequent ultrasound demonstrated air within the spinal canal. A PICC line was placed in the right arm for IV access and the infant was transferred to our facility.

**Figure 2:**
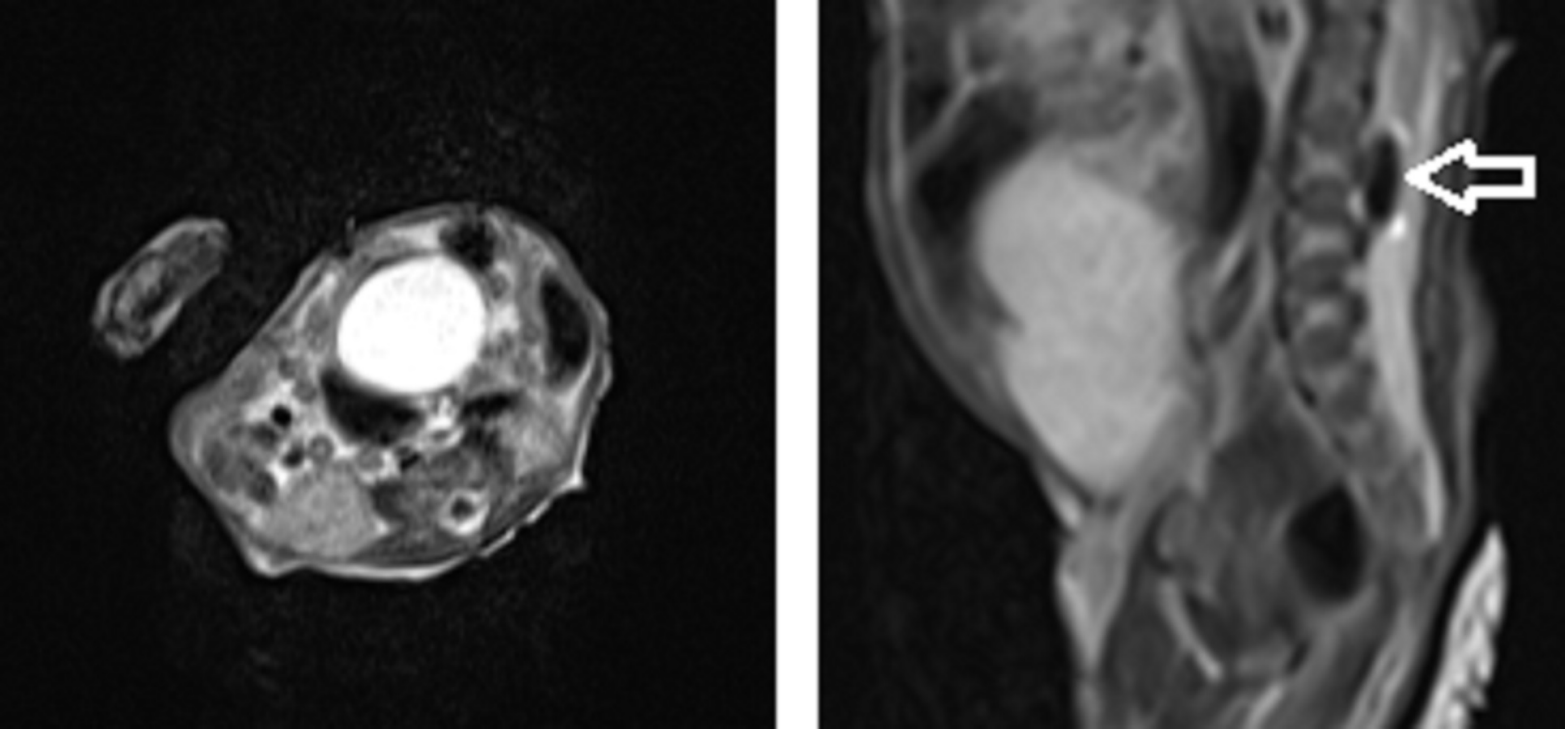
MRI of Lumbar Spine Sagittal T2 (Right) and axial T2 (Left) sequence, respectively, demonstrating a hypointense lesion in the anterior thecal sac extending from L2-L3 consistent with a large air bubble (Arrow)

**Figure 3:**
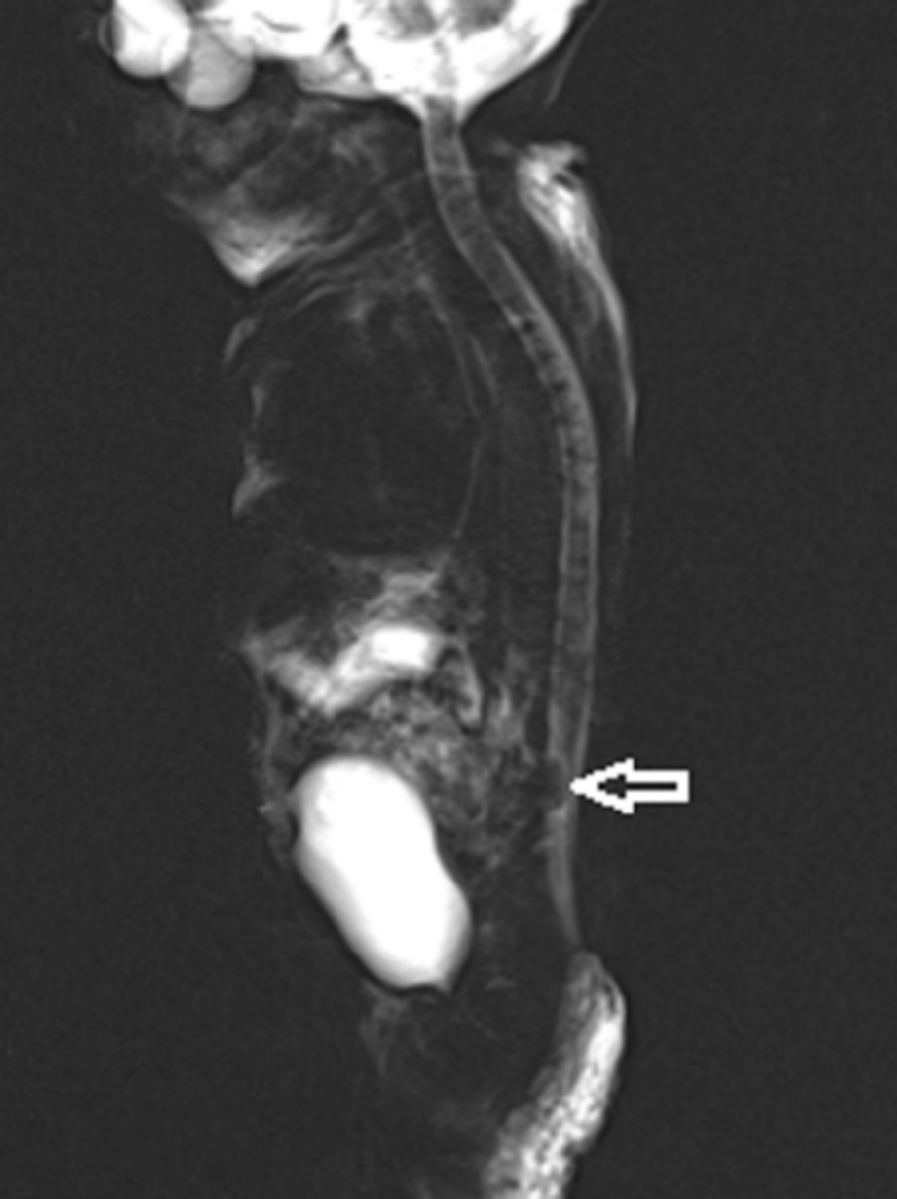
3-D Myelogram Image Further demonstrating the hypointense large air bubble within the thecal sac in the lumbar region (Arrow)

Upon neurosurgical evaluation in the Penn State Hershey Children’s Hospital NICU, the infant was found to be moving all extremities spontaneously and against gravity but with comparatively diminished strength in the left lower extremity. A magnetic resonance imaging (MRI) scan of the brain and spine was performed on DOL four and showed signal dropout on T1 and T2 weighted images in the anterior spinal canal posterior to L2-L3 (Figures 2-3). There was no evidence of gross cord compression. There were no other abnormalities found.

Subsequent physical examinations found that the infant’s left lower extremity weakness gradually improved without intervention until it was equal in strength to the right side. A follow-up ultrasound of the lumbar spine on DOL five showed resolution of the air within the spinal canal.

## Discussion

Pneumorrhachis, defined as the presence of air within the spinal canal, can be separated into intradural and extradural subtypes. PR can additionally be classified as primary or secondary depending on whether it is the cause or the result of air in other body compartments [7]. The etiology of PR is varied. Case reports attribute it to trauma, high intrathoracic pressure and barotrauma, iatrogenic manipulation, malignancy and violent coughing [8]. It is, almost without exception, found in combination with air in other compartments of the body and is most commonly associated with pneumocephalus, pneumothorax, pneumomediastinum, pneumopericardium or subcutaneous emphysema. Other case reports describe it as a complication of administration of epidural anesthesia or placement of an epidural blood patch [9-10]. Most patients are asymptomatic and the condition is self-limited, with reabsorption of the air occurring spontaneously [7]. However, there have been case reports of PR leading to neurological symptoms and/or deficits due to compression of the spinal cord and/or nerve roots [10]. In some cases, surgical intervention was necessary. 

## Conclusions

We report a novel presentation of isolated pneumorrhachis occurring after inadvertent insertion of a PICC line into the ascending lumbar vein. This resulted in transient lower extremity strength deficits which resolved over time and correlated with the disappearance of air within the spinal canal on subsequent radiographic studies.
